# Extracting Wetland Type Information with a Deep Convolutional Neural Network

**DOI:** 10.1155/2022/5303872

**Published:** 2022-05-18

**Authors:** XianMing Guan, Di Wang, Luhe Wan, Jiyi Zhang

**Affiliations:** ^1^Heilongjiang Province Key Laboratory of Geographical Environment Monitoring and Spatial Information Service in Cold Regions, Harbin Normal University, Harbin 150025, China; ^2^College of Geographic Science, Harbin Normal University, Harbin 150025, China; ^3^Heilongjiang Geomatics Center of NASMG, Harbin 150081, China; ^4^Heilongjiang Institute of Geomatics Engineering, Harbin 150081, China; ^5^College of Geographic Science, Nantong University, Nantong 226019, China; ^6^Department of Geographic Information Science, Chuzhou University, Chuzhou 239000, China

## Abstract

Wetlands have important ecological value. The application of wetland remote sensing is essential for the timely and accurate analysis of the current situation in wetlands and dynamic changes in wetland resources, but high-resolution remote sensing images display nonobvious boundaries between wetland types. However, high classification accuracy and time efficiency cannot be guaranteed simultaneously. Extraction of wetland type information based on high-spatial-resolution remote sensing images is a bottleneck that has hindered wetland development research and change detection. This paper proposes an automatic and efficient method for extracting wetland type information. First, the object-oriented multiscale segmentation method is used to realize the fine segmentation of high-resolution remote sensing images, and then the deep convolutional neural network model AlexNet is used to classify automatically the types of wetland images. The method is verified in a case study involving field-measured data, and the classification results are compared with those of traditional classification methods. The results show that the proposed method can more accurately and efficiently extract different wetland types in high-resolution remote sensing images than the traditional classification methods. The proposed method will be helpful in the extension and application of wetland remote sensing technology and will provide technical support for the protection, development, and utilization of wetland resources.

## 1. Introduction

With the development of remote sensing technology, the quality of available remote sensing images is increasing, and information on ground objects is becoming increasingly detailed; however, the classification accuracy of the traditional pixel-based classification method is low due to the large amount of data, and it can no longer meet the classification requirements of high-resolution remote sensing images [1]. Shallow structure models, such as neural networks and support vector machine ensembles, combine spectral, structural, and semantic features for the classification of high-resolution remotely sensed imagery [2]. However, due to the high resolution of remote sensing images, the heterospectrum phenomenon is very obvious. For complex sample structures, shallow structure models are affected by computing power and are unable to learn the sample information adequately [3]. A deep learning network is a deep structure model composed of multiple nonlinear mappings, has a strong function expression ability, can learn more complex training samples, and has good robustness for the classification of complex features in remote sensing images [4, 5].

Wetlands play an important role in regulating the natural environment; they are considered a unique ecosystem with rich biodiversity formed between the interaction between water and land, and they have important ecological value [6, 7]. The status and dynamic changes of wetland resources can be accurately studied in a timely manner by using high-precision and high-spatial-resolution remote sensing image classification methods, which are of great significance in wetland research [8]. However, the boundary between wetland types in high-resolution remote sensing images is not obvious; at present, automatic and efficient extraction of wetland type information has been a bottleneck hindering wetland development and change monitoring [9, 10].

The traditional supervised classification algorithm of remote sensing image based on pixel classifies mainly the image according to the ground object spectrum, which depends on the priori knowledge and practical experience [11]. The diversity of spectral information brings challenges to the high-resolution image classification. The traditional classification method based on physical model and statistical model has been difficult to apply in the remote sensing information extraction in the era of big data [12, 13]. Due to the differences of ground object targets, the neural network needs to extract effectively the features of ground object targets under different receptive fields. Deep learning semantic segmentation based on pixel classification can analyze quickly the deep semantic information of image and has become the most advanced technology in the field of image segmentation [14, 15].

With the improvement of computer technology, the classification method of wetland remote sensing has also been continuously developed. Decision tree has the characteristics of flexibility, intuition, and high efficiency, and it has been widely used in grassland wetland evaluation and freshwater swamp information extraction [16]. In the 1980s, the classification method such as neural network [17] and support vector machine [18] was gradually applied in the classification of wetland remote sensing images. These models can improve the classification accuracy of high-resolution remote sensing images. However, for the complex sample structure, the shallow structure model is affected by the computing power and cannot fully learn the sample information. But the deep structure model can make full use of the spatial structure information of the image by classifying the high-resolution wetland remote sensing image. The convolutional neural network extracts suitably the input features layer by layer from the low level to the high level to form the network weight structure, which is more suitable for dealing with complex ground object classification. More and more scholars introduce convolutional neural network to deal with the problems of feature extraction, classification, and scene recognition of high-resolution remote sensing images, but the research on wetland classification system is very rare.

By outlining classification requirements for extracting wetland type information, this paper presents a method to automatically and efficiently extract wetland type information based on high-spatial-resolution remote sensing images and verifies the classification results by specific experimental cases. By comparing the proposed method with traditional classification methods in terms of extraction precision and efficiency, the high accuracy and efficiency of the method are suggested, and the possibility of accurately and efficiently extracting wetland type information is provided.

## 2. Basic Idea

**Figure 1:**
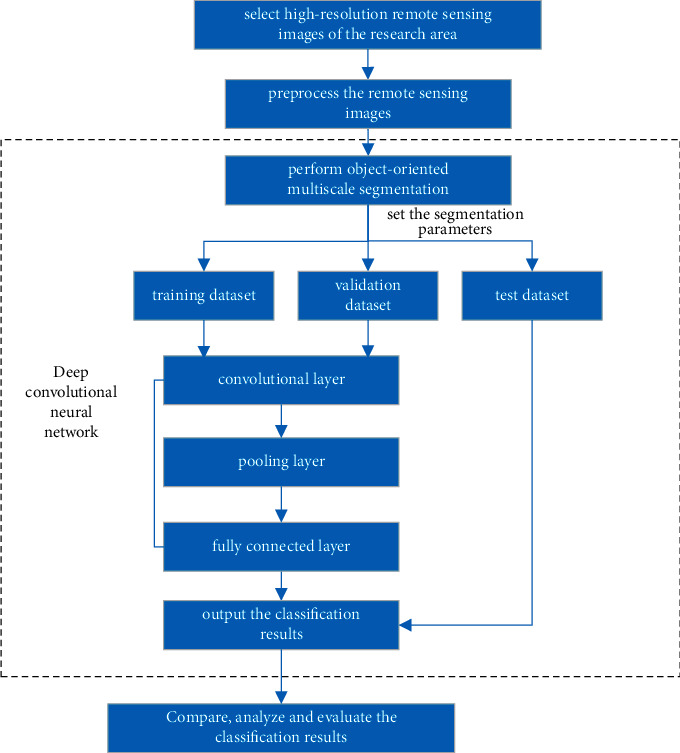
Technology guidelines.

Wetlands are considered an important natural resource, and the emergence of high-resolution remote sensing images in recent years has provided a basic condition for large-scale wetland information extraction; by analyzing comprehensively the existing methods, it is possible to improve the accuracy and efficiency. This paper selects an area with rich wetland types as the research area. Based on high-resolution remote sensing image data, the object-oriented method is used to segment the system. A deep convolutional neural network model is used to extract wetland type information, and taking field-measured data as the reference for accuracy verification, the extracted results are compared with traditional classification methods, and an automatic and efficient method for extracting wetland type information is proposed. The method is as follows (Figure 1):

1. Original high-spatial-resolution remote sensing images in the study area were selected, and the radiometric geometric correction images were preprocessed with methods such as fusion, stitching, and clipping. The distribution characteristics of the remote sensing image features were analyzed by a visual interpretation method, and the wetland type classification system in the study area was established based on the field-measured data.
2. After preprocessing the remote sensing images for object-oriented multiscale segmentation and standardizing the segmentation of the data, the dataset was divided into a training set, validation set, and test set according to a ratio of 3 : 1 : 1. A deep convolutional neural network was trained based on the depth of the model to achieve the extraction of feature information by the high-resolution remote sensing images.
3. The iterative self-organizing data analysis technique (ISODATA) algorithm, maximum likelihood, and backpropagation (BP) neural network classification methods were selected to classify the remote sensing images in the study area, and the extraction results of the proposed method were compared with those of the traditional classification methods. In addition, the accuracy of the results of each of the models was evaluated, and the advantages of deep convolutional neural networks for extracting wetland type information from high-resolution remote sensing images were determined.

### 2.1. Object-Oriented Multiscale Segmentation

The object-oriented multiscale remote sensing image segmentation method is a kind of image feature information extraction technology for the classification of high-resolution remote sensing images [19, 20]. Different from traditional remote sensing image classification methods based on pixels, the object-oriented method divides images into objects according to certain homogeneity and spectral texture structures [21]. The smallest unit is no longer a pixel but an image object. In the segmentation process, similar pixels are divided into the same object, and adjacent objects have obvious differences. The object-oriented method can obtain segmentation objects with relatively regular edges with high spatial accuracy, and the classification results have a certain integrity, which can avoid the occurrence of salt-and-pepper noise [22].

Multiscale segmentation technology is often used in the classification of remote sensing images, and the image segmentation effect is directly affected by the scale parameters. For example, if a large area of image objects is obtained, a large-scale parameter is set, and the number of objects is small correspondingly. When the scale parameters and compactness parameters are fixed, different shape parameters have different effects on the segmentation results. The larger the shape parameter, the more regularly segmented the object; the smaller, the more irregular. In the multiscale segmentation of remote sensing images, the selection of segmentation scale and corresponding segmentation parameters should be based on the image feature. The segmentation scale and parameters corresponding to different feature are different [23]. In this paper, the segmentation scale and corresponding parameters are selected through repeated experiments.

### 2.2. AlexNet Convolutional Neural Network

**Figure 2:**
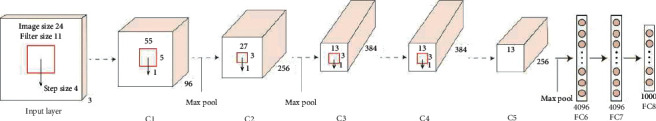
Schematic diagram of AlexNet.

The AlexNet model is a typical deep convolutional neural network model; in recent years, it has achieved breakthroughs in the field of image recognition [24, 25]. The AlexNet model obtains the original image characteristics in the input layer and the filter with the sample characteristics. Through 5 convolutional layers and 3 fully connected layers, the depth model with an 8-layer network structure is obtained, which achieves efficient training and has a stable convergence rate. Compared with AlexNet model, other deep convolutional neural network models, such as VGGNet, GoogLeNet, ResNet, and so on, all have far more layers than AlexNet. With the increase of layers, it will bring huge parameter calculation. With the increase of neural network depth, the accuracy of the model will first rise and be saturated. When the depth continues to increase, the accuracy will decline. Because with the increase of the number of layers, there will be gradient explosion or attenuation, the gradient will become unstable, and the value will be particularly large or small. Therefore, the network performance will become worse and worse. Too many parameters lead to excessive memory consumption and excessive computing resources. Although MobileNet and other methods with less layers have less floating-point calculation, networks at different levels can learn the characteristics of different levels. With the reduction of layers, the ability to mine the detailed information of ground objects is affected, and the classification accuracy is reduced [26]. AlexNet uses ReLU activation function to solve the gradient dispersion problem and improve the training speed when the network is deep; dropout is used in training to avoid overfitting. LRN (local response normalization) is used to enhance the generalization ability of the model and improve the accuracy of training. In addition, based on GPU, AlexNet uses CUDA to accelerate the training of neural network and improve the training efficiency of the network.  Figure 2 shows the schematic diagram of the AlexNet model.

## 3. Case Study

### 3.1. Selection of the Experimental Data

**Figure 3:**
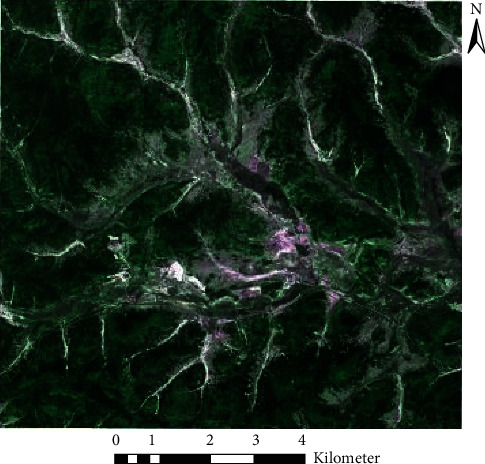
True color image of the study area.

We selected remote sensing images of the Heilongjiang Gongbiela River National Nature Reserve taken by the Gaofen-2 (GF-2) satellite as the experimental data. Panchromatic and corresponding multispectral imaging data of two scenes were collected in July 2018 to obtain a mosaic remote sensing image with a spatial resolution of 1 m after preprocessing. The data are shown in Figure 3.

### 3.2. Image Multiscale Segmentation

**Figure 4:**
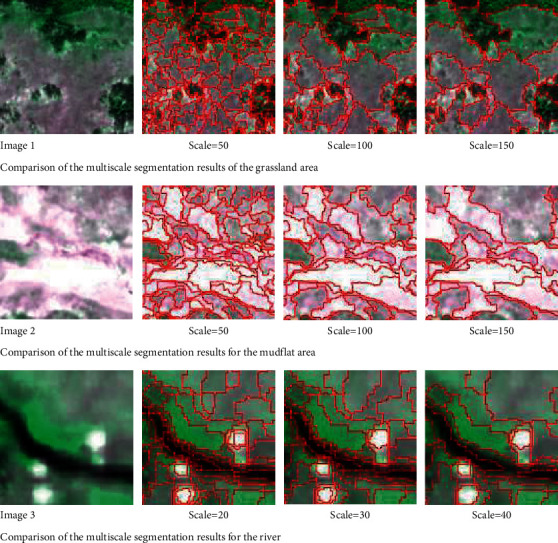
Multiscale segmentation of wetland type information.

According to visual interpretation, the main wetland types in the preliminary study area were determined. After repeated comparative analysis during image segmentation, the wetland types were first segmented at multiple scales. The boundary of the segmentation objects is more accurate when the segmentation scale parameters of the grassland and mudflat areas (layer 1) are 100. The boundary of the river (layer 2) is more accurate when the segmentation scale parameter is 30. On the condition that the segmentation scales of the above two layers remain unchanged, the weight of the compactness parameters is fixed, and the weight is gradually increased from 0.1 to 0.9 according to an arithmetic sequence to determine the shape parameters. Based on this experiment, layer 1 achieves a better segmentation effect on grassland and mudflat when the segmentation scale parameter is 100, the shape parameter is 0.4, and the compactness parameter is 0.6. Layer 2 is taken as the sublayer of layer 1 and has a better segmentation effect on rivers when the segmentation scale parameter is 30, the shape parameter is 0.3, and the compactness parameter is 0.7. The multiscale segmentation parameters for extracting wetland type information in the study area are shown in Table 1. The multiscale segmentation results are shown in Figure 4.

Similarly, the cultivated land area was divided according to a segmentation scale parameter of 100, a shape parameter of 0.4, and a compactness parameter of 0.6. The remaining images to be segmented were forestland (since the road class has a maximum width of 6 pixels and is not considered as a research focus, it is classified as the adjacent feature type).

**Figure 5:**
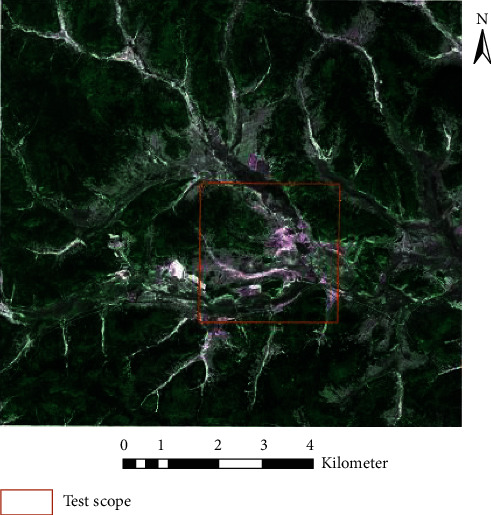
Test scope.

**Figure 6:**
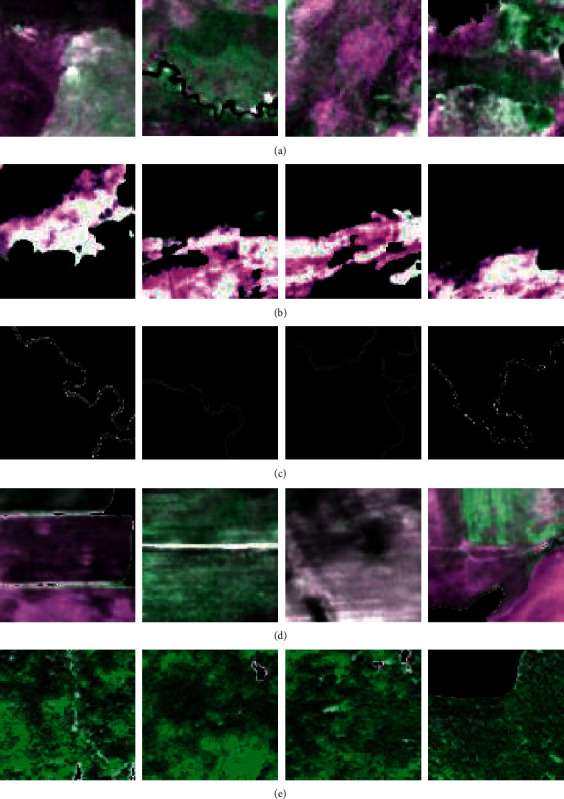
Examples. (a) Grassland. (b) Mudflat. (c) River. (d) Cultivated land. (e) Forest.

The segmented wetland images were converted into three-channel red, green, and blue (RGB) images, and the segmented images were divided into three standard datasets of 256 ×× 256 images, namely, a training set, verification set, and test set, according to a ratio of approximately 3 : 1 : 1. A typical area of the research area was selected as the test area, and the segmented images were produced into a standard test set. The scope of the test area is shown in Figure 5. The image segmentation data outside the test area were divided into a standard training set and validation set. There were 3224 samples in the training set, 908 samples in the verification set, and 969 samples in the test set (test areas). Examples of the training set, verification set, and test set are shown in Figure 6. Part of the masked RGB values of the samples are filled in with 0 s.

### 3.3. Extraction of the Wetland Type Information

**Figure 7:**
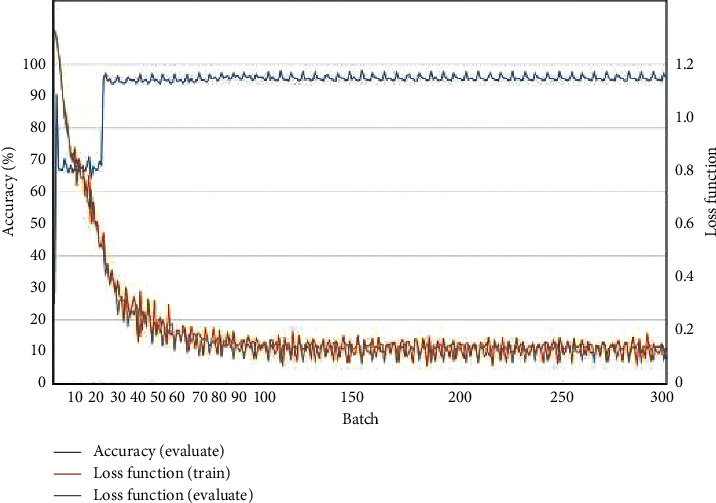
Accuracy and loss function curves.

**Figure 8:**
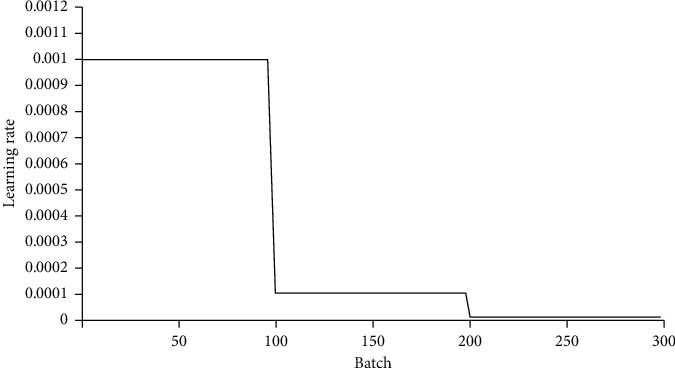
Rate-of-change curve in the training stage.

In this study, the Caffe machine learning framework and AlexNet convolutional neural network are applied for wetland type information extraction. During the training process, some neurons were randomly deleted in each iteration, and softmax logistic regression was applied to classify the images in the output layer. The initial value of the learning rate was set to 0.001. The deep convolutional neural network was trained and evaluated through 5 types of land cover types, namely, grassland, mudflat, river, cultivated land, and forest, so there were 5 outputs in the fully connected layer. The network is optimized to obtain the multivariate logistic regression model of the maximum average. The network training process is shown in Figures 7 and 8.

It can be seen from the experiment that the AlexNet deep convolutional neural network model achieves a good effect on the experimental dataset after effective training and learning. The accuracy of batches 0 to 20 was unstable, with an initial accuracy of 25.2%; the accuracy rapidly increased to 90.4%, then rapidly decreased to 67.2%, and stabilized at approximately 70%. After 20 batches, the accuracy began to rise gradually, and the highest accuracy achieved was 97.2%. After 100 batches, the accuracy reached a high level and stabilized. At the same time, the initial loss function was approximately 1.1, and the loss function of batches 0 to 20 rapidly converged to approximately 0.4. The loss function gradually began to converge after 20 batches. The loss function of batch 100 was less than 0.1, and the convergence effect was good and tended to be stable. As the loss function converges rapidly and tends to be stable, the accuracy increases rapidly and remains high. The initial learning rate was 0.001, which gradually decreased to 0.0001 after 100 batches and then continued to decline and gradually stabilized after 200 batches, indicating that the deep convolutional neural network achieves an ideal learning effect on the dataset.

### 3.4. Analysis and Verification of the Results

**Figure 9:**
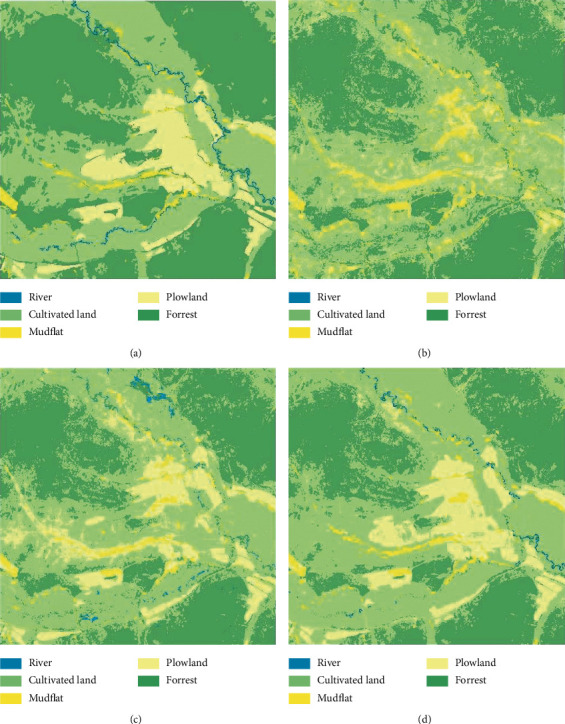
Classification results. (a) Classification results of the method proposed in this paper. (b) Classification results of the ISODATA algorithm. (c) Classification results of the maximum likelihood method. (d) Classification results of the BP neural network.

This research uses field-measured data and visual interpretation results to verify the effectiveness of the convolutional neural network model for the wetland classification, and its accuracy and efficiency in extracting wetland type information were compared with those of traditional methods, namely, an unsupervised classification method (the ISODATA algorithm), a supervised classification method (the maximum likelihood (ML) method), and shallow machine learning classification method (a BP neural network) on the same data. The classification results of the test set images by the proposed method and the three traditional methods are shown in Figure 9.

All the results of the traditional remote sensing image classification methods, that is, the ISODATA algorithm, ML, and BP neural network, have “salt-and-pepper noise” [27–29]. However, the classification method selected in this paper obtained clear features. After about fifteen repeated experiments, the following indexes are selected to evaluate the accuracy and efficiency of the methods: the producer's accuracy, user's accuracy, overall accuracy, kappa coefficient, and classification time. The classification accuracy and statistical results are shown in Table 2.

Through the comparative analysis of the classification results of the four methods, it can be seen that for the extraction of wetland type information from the high-resolution remote sensing images, the identification accuracy of the four classification methods is higher for the grassland and mudflat areas, which is due to the large difference in the image characteristics of these land cover types. Although the river image has obvious spectral features, the ISODATA algorithm fails to extract the river information effectively and automatically classifies it into the surrounding features because the local width is no more than 10 pixels. The maximum likelihood classification method can extract the river information, but the river is relatively fragmented. The river features extracted by the BP neural network and deep convolutional neural network are relatively consistent, and the deep convolutional neural network achieves the best river connectivity effect. Compared with the four classification methods, the maximum likelihood method and the deep convolutional neural network method are faster; they take 6 minutes and 4 minutes, respectively. However, the overall classification accuracy of the maximum likelihood method is low. Although the ISODATA algorithm does not need to understand the research area in advance, reduces the interference of human factors, and achieves a moderate classification time (26 minutes), it misclassifies and fails to classify many areas, and its overall classification accuracy is the lowest; therefore, it does not meet the requirements of classification. Although the overall accuracy of the BP neural network classification method is high, it takes 185 minutes to run, which is too long for practical applications. Based on the above analysis, it can be concluded that the deep convolutional neural network can quickly and effectively identify wetland type information, with high classification accuracy, short runtime, and ideal effect.

## 4. Conclusion and Discussion

At present, the status and dynamic changes of wetland resources cannot be determined in a timely and accurate manner by the current wetland remote sensing method. This paper proposes an automatic method that combines application of object-oriented multiscale segmentation technology and a deep convolutional neural network based on remote sensing image feature extraction technology. This paper extracts wetland type information from high-resolution remote sensing images of the Heilongjiang Gongbiela River National Nature Reserve. The final results are verified and compared with the traditional methods. The results show that the deep convolutional neural network can extract wetland types from remote sensing images better than the traditional methods; for example, the deep convolutional neural network can handle nonobvious boundaries better than the traditional methods. Depth characteristics can be better expressed, and more detailed information of wetland types can be mined, which not only improves the classification accuracy but also substantially improves the extraction efficiency compared with the traditional classification algorithms. The research method proposed in this paper achieves a higher accuracy and has a wider applicability than the traditional methods. However, as a new classification technology, convolutional neural network has broad research space. Based on the results of remote sensing image processing in different regions, this study has still partial limitations and deficiencies, which will be further improved in the future research.

Next, the authors will focus on the GoogLeNet [30], Visual Geometry Group (VGG) [31, 32], and ResNet convolutional neural network architectures and the advantages and disadvantages of neural network models for extracting wetland types from high-resolution remote sensing images. This article created a large number of sample datasets according to the types of features and characteristics of the remote sensing data sources; however, to address a large number of wetland types for environment information extraction from remote sensing images, future research will also increase the number of datasets and diversify the types of datasets, expand the study area, and improve the generalization ability of the model. How to select the optimal parameters and improve the classification efficiency needs the further study.

## Figures and Tables

**Table 1:** Multiscale segmentation parameters for extracting wetland type information.

Wetland type	Scale parameter	Shape parameter	Compactness parameter	Smoothness parameter	Weights
Grassland	100	0.4	0.6	0.4	1, 1, 1, 1
Mudflat	100	0.4	0.6	0.4	1, 1, 1, 1
River	30	0.3	0.7	0.3	1, 1, 1, 1

**Table 2:** Comparison of the classification accuracy.

Precision	ISODATA algorithm		Maximum likelihood method		BP neural network		Deep convolutional neural network	
	Producer's accuracy	User's accuracy	Producer's accuracy	User's accuracy	Producer's accuracy	User's accuracy	Producer's accuracy	User's accuracy
River	0.00	0.00	53.23	100.00	66.13	100.00	74.19	100.00
Grassland	87.30	64.45	90.48	69.51	91.01	79.26	95.77	90.95
Mudflat	90.98	93.08	90.23	97.56	91.73	99.19	90.91	99.17
Cultivated land	54.17	49.06	72.92	55.56	87.50	58.33	91.67	65.67
Forest	66.06	68.99	72.12	90.15	86.67	99.31	97.56	98.77

Overall accuracy	70.5193%		80.0670%		87.1022%		92.6050%	
Kappa coefficient	0.5991		0.7336		0.8293		0.9022	
Time (minutes)	26		6		185		4	

## Data Availability

The data used to support the findings of this study are available from the corresponding author upon request.
